# Supplementary material for: Short-Chain Fatty Acids Modulate Sperm Migration through Olfactory Receptor 51E2 Activity
Source: Int J Mol Sci. 2022 Oct 22;23(21):12726. doi: 10.3390/ijms232112726 (PMC9658011; doi:10.3390/ijms232112726)
Supplement: Supplementary file 1 [file ijms-23-12726-s001.zip › ijms-1981906-supplementary.pdf]

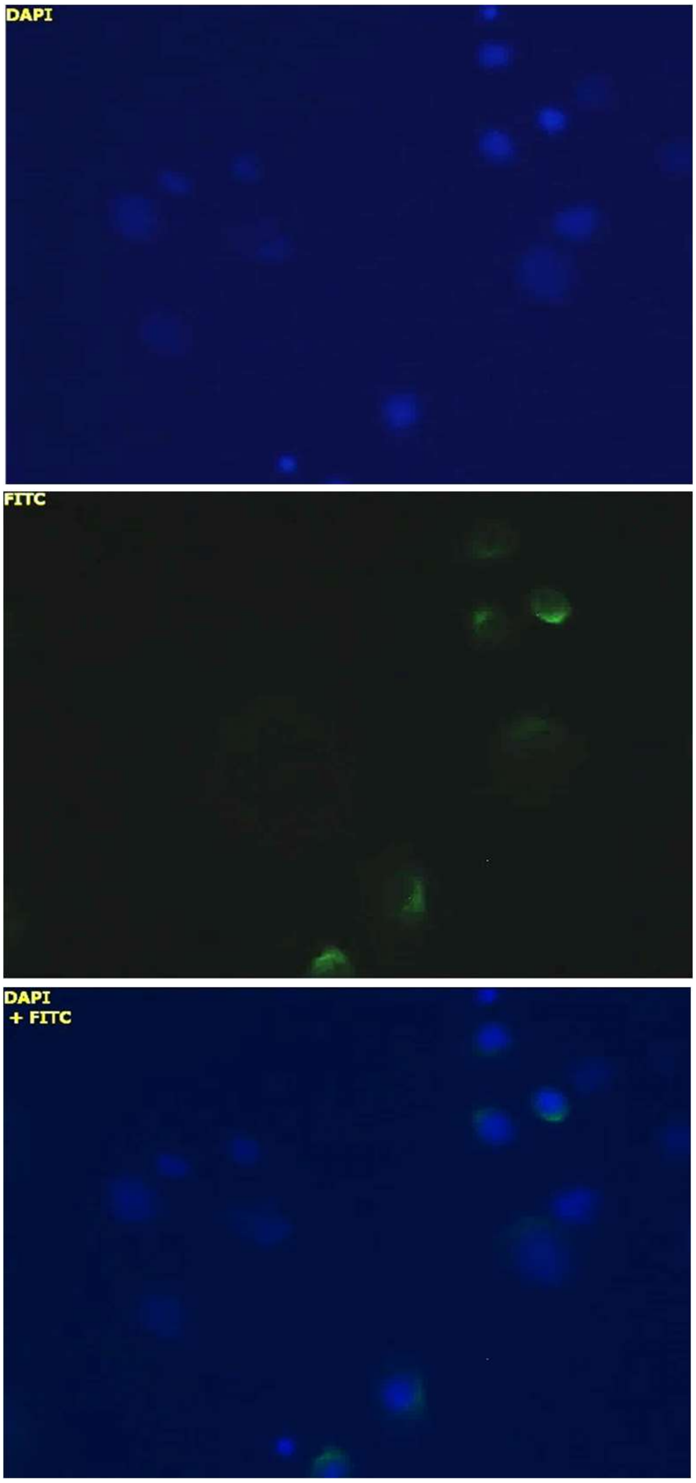

Figure S1. Overexpression of FLAG-Rho-OR51E2 in Hela cells
Representative pictures of immunofluorescence analysis of OR51E2 (green). Nuclei were counterstained with DAPI (blue). The lowest panel show the merged signal between OR51E2 and nuclei.

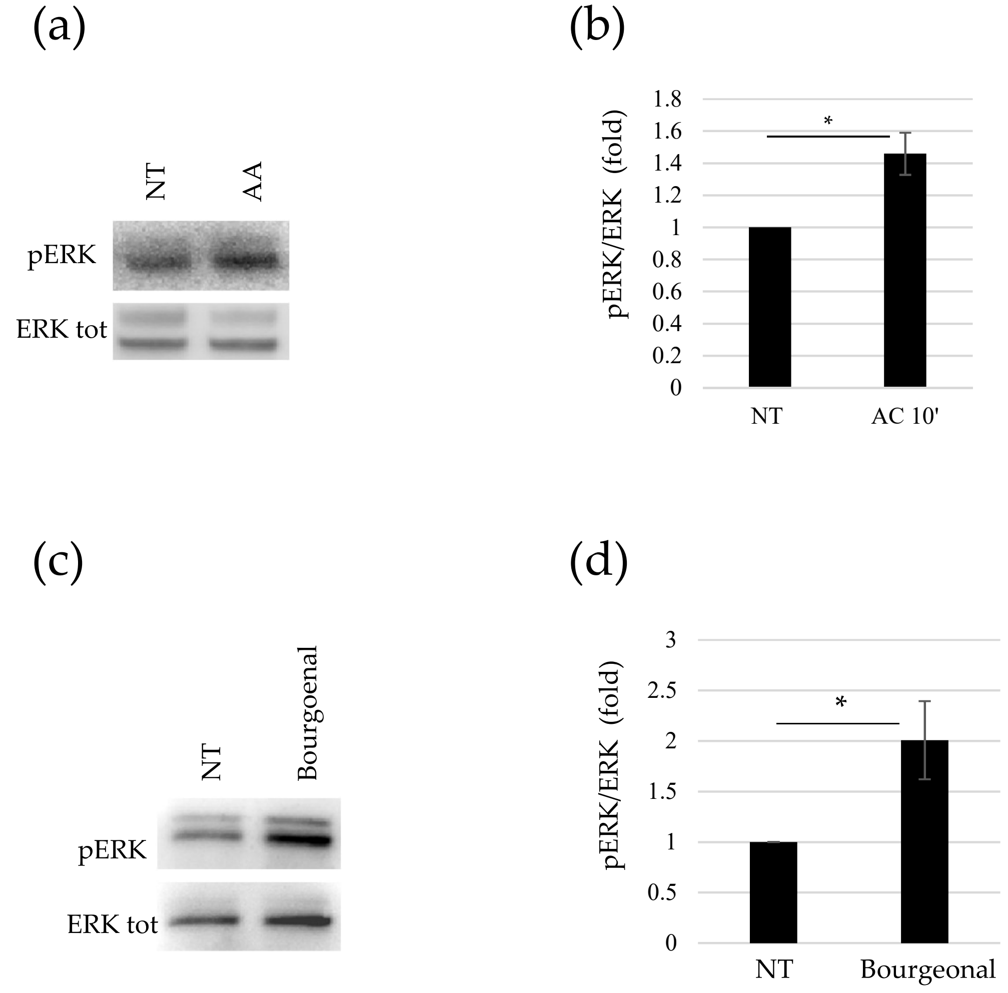

Figure S2. Activation of spermatozoa by Acetic Acid (AA) and Bourgeonal
(a) Representative western blot (WB) analysis of pERK and total ERK protein in sperm cells treated with 5mM AA for 10 minutes. (b) Histogram shows the ratio of densitometric values of pERK to total ERK after AA treatment. The densitometric value of untreated sperm cells (NT) is arbitrarily set to 1. Mean ± SD of three independent biological replicates is shown (N = 3. *=$p < 0.05$, one sample t-test). (c) Representative western blot (WB) analysis of pERK and total ERK protein in sperm cells treated with 500 μM Bourgeonal for 10 minutes. (**D**) Histogram shows the ratio of densitometric values of pERK to total ERK after Bourgeonal treatment. The densitometric value of untreated sperm cells (NT) is arbitrarily set to 1. Mean ± SD of three independent biological replicates is shown (N = 3. *=$p < 0.05$, one sample t-test).

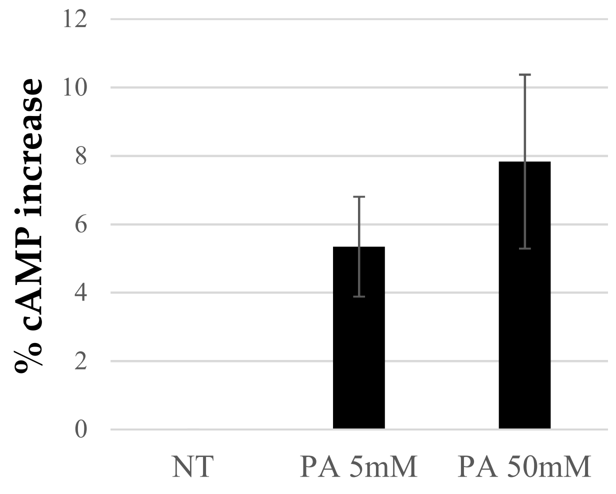

Figure S3. Activation of Adenylate cyclase by PA
Histogram shows the % of increase of Cyclic adenosine 3′,5′-monophosphate (cAMP) in sperm cells treated with the indicated doses of PA for 15 min compared to untreated cells (NT). Mean ± SD of two independent biological replicates is shown.
